# Dipolar relaxation mechanism of long-lived states of methyl groups

**DOI:** 10.1007/s11128-017-1777-6

**Published:** 2017-12-09

**Authors:** Razieh Annabestani, David G. Cory

**Affiliations:** 1 Institute for Quantum Computing, University of Waterloo, Waterloo, ON N2L 3G1 Canada; 2 Department of Physics and Astronomy, University of Waterloo, Waterloo, ON N2L 3G1 Canada; 3 Department of Chemistry, University of Waterloo, Waterloo, ON N2L 3G1 Canada; 4 Perimeter Institute for Theoretical Physics, Waterloo, ON N2L 2Y5 Canada; 5 Canadian Institute for Advanced Research, Toronto, ON M5G 1Z8 Canada

**Keywords:** Relaxation, Symmetry, Long-lived states

## Abstract

We analyze the symmetry properties of the dipolar Hamiltonian as the main relaxation mechanism responsible for the observed NMR spectra of long-lived states of methyl groups. Long-lived states exhibit relaxation times that are considerably longer than the spin–lattice relaxation time, $T_{1}$. The analysis is complementary to previous studies and provides insight into the relaxation mechanism of long-lived states by focusing exclusively on the symmetry of the spin Hamiltonian. Our study shows that the dipole–dipole coupling between protons of a methyl group and between the protons and an external spin are both symmetry breaking interactions that can lead to relaxation pathways that transform the polarization from symmetry order to Zeeman order. The net contribution of the internal dipolar interaction to the NMR observation of long-lived states is zero. Our calculation is in good agreement with the reported features of the observed spectra and previous theoretical studies.

## Introduction

Implementing states with long relaxation times is an attractive approach to quantum memories, sensors in quantum meteorology and as a tool for studies of slow processes in nuclear magnetic resonance (NMR) [1–4].

NMR is normally concerned with states polarized along the external magnetic field at some temperature. Thus, the spin–lattice interaction is the dominant source of energy relaxation with its characteristic time, $T_{1}$. There are, however, alternative ways of polarizing the spin system such that the polarized state is protected from the majority of relaxation mechanisms, allowing the relaxation time to substantially exceed $T_{1}$. For instance, a group of indistinguishable spins have symmetry properties that are preserved under collective noise processes. These symmetry degrees of freedom can be used for implementing long-lasting states [5–7].

Long-lived states (LLSs) have been implemented experimentally [6–15] and studied theoretically [16–19]. For the simplest case of two spin $\frac{1}{2}$ particles, the four spin eigenstates can be categorized into two symmetry groups: triplet states and singlet state. The former is invariant under spin exchange and the later is antisymmetric. By creating an imbalance of population between the singlet state and the identity in the triplet subspace, one can prepare a *symmetry-polarized* state that exhibits long relaxation time due to its resistance to the majority of relaxation mechanisms [8, 9]. One can extend this idea to three identical spins, where the eight eigenstates are categorized into three groups labeled *A*, $E_{+}$ and $E_{-}$ where the last two are degenerate subspaces. Under cyclic permutation of the spins, the four eigenstates in group *A* remain invariant, and so, *totally symmetric* states, whereas the two eigenstates in each of degenerate subspaces, $E_{\pm }$, acquire a phase $\varepsilon = \hbox {e}^{\pm \frac{2 \pi }{3}}$, and so, *non-symmetric* states [12, 17, 20, 21]. In a similar manner to the two-spin case, one can polarize three identical spins with respect to their symmetry by creating an imbalance of population between the *A* states and the $E_{\pm }$ states. This has been implemented in methyl groups, and the relaxation time has been extended by factor of 7 beyond the single spin $T_{1}$ [12].

The LLS is immune to collective noise because the symmetry of the states is also collective. Although the symmetry is preserved, the magnetization is not. Consider a state of three identical spins that have both symmetry order and magnetization order.^1^ In the absence of any symmetry breaking interaction and under a collective depolarizing noise, that system can evolve to a state with no magnetization order. Therefore, no signal of that state can be observed in NMR. However, one can still observe a signature of the symmetry order. In fact, any interaction that does not preserve the permutational symmetry of spins, in principle, can lead to a relaxation process that turns the polarization from *symmetry order* into *Zeeman order*, converting an initially prepared LLS to an NMR observable state. For example, in [12], accessing the noise-protected subspaces has been achieved at low temperature where the majority of those interactions that distinguishes spins are *frozen* or have negligible effect. On the contrary, the NMR observations have been done at room temperature where those spin distinguishing relaxation processes are no longer negligible. A few examples of such interactions are the chemical shift anisotropy (CSA) and the dipole–dipole coupling between spins. The authors of [17] have studied the contribution of both of these interactions on the NMR observation of LLS in spherical top molecules. They derive the NMR spectra in the limit of relatively short correlation time of methyl groups. Here, we describe an approach to calculate the contribution of the dipole–dipole interaction to the NMR observation of long-lived states, which focuses exclusively on the symmetry properties of the dipolar Hamiltonian. Our study is complementary to previous studies [12, 17].

The spectrum of initially prepared LLS in methyl groups has been reported and has some unique features [12, 21] that differentiate an initial symmetry-polarized state from a thermally polarized state. The scalar coupling between the methyl group with total spin operator $\mathbf S = \frac{1}{2} \sum \nolimits _{i=1}^{3}\ \overrightarrow{\sigma }^{H}_{i}$ and the carbon with spin operator $\mathbf {I}= \frac{1}{2} \overrightarrow{\sigma }^{C}$ is given by $2\pi \ J_{HC} \ \mathbf S \ .\ \mathbf {I}$, which leads to splitting the proton and the carbon spectra into multiple peaks. On the proton channel, one would observe two resolved peaks, $\langle S_{z} \otimes |\uparrow \rangle \langle \uparrow |\rangle$ and $\langle S_{z} \otimes |\downarrow \rangle \langle \downarrow |\rangle$, each corresponding to the carbon’s state being aligned or anti-aligned with the field. On the carbon channel, one would observe four resolved peaks, $\langle \varPi _{\pm \frac{ 3}{2}} \otimes I_{z}\rangle$ and $\langle \varPi _{\pm \frac{ 1}{2}} \otimes I_{z}\rangle$, each corresponding to the total magnetization of the methyl group being $\pm \frac{3}{2}$ or $\pm \frac{1}{2}$. The explicit definition of $\varPi _{\pm \frac{ 3}{2}/\frac{ 1}{2} }$ is given in Eq. 33. In case of symmetry-polarized states, the two peaks on the proton channel have equal amplitudes but with opposite phases. The four peaks on the carbon channel have different amplitudes, and they are two-by-two anti-phase with one another [12, 22], as opposed to a thermally polarized state where all peaks on both spectra are in-phase with each other. In this report, we analytically calculate the NMR spectra of an initially prepared LLS and predict the above- described features that have been observed. Our result is consistent with the results in [12, 17]. Our approach relies on the symmetry properties of the dipolar Hamiltonian and explains the underlying physics that leads to extended relaxation time and observed features of the NMR spectra.

In Sect. 2, we review the definition of long-lived states in the case of three indistinguishable spins. In Sect. 3, we expand both the heteronuclear and homonuclear dipolar Hamiltonians in terms of symmetrized spin operators and analyze their properties. The symmetry analysis allows us to solve the master equation analytically in Sect. 4 and to predict the NMR spectrum of LLS in methyl groups. We report a summary of this work in Sect. 5.

## Long-lived state in methyl groups

In the presence of a uniform static magnetic field, $B_{0}$, the spin Hamiltonian of protons in a methyl group is

1 $$\begin{aligned} H_{\text {spin}}= \frac{\omega _{h}}{2} \sum \limits _{i=1}^{3} \ \sigma ^{(i)}_{z} + 2\pi \ J_{0}\ \sum \limits _{j< k} \ \overrightarrow{\sigma }^{(j)}.\overrightarrow{\sigma }^{(k)} + H_\mathrm{CSA} + H_\mathrm{DD}, \end{aligned}$$

where $\omega _{h}= \gamma _{h}B_{0}$ is the proton frequency, $J_{0}$ is the scalar coupling constant between any two protons and $H_\mathrm{CSA}$ and $H_\mathrm{DD}$ account for the chemical shift anisotropy (CSA) and the dipole–dipole (DD) interaction between any two spins. At relatively large field, when the Zeeman interaction is the dominant term in the Hamiltonian, it is a good approximation to treat these protons as three indistinguishable spins. The spin eigenstates are

2 $$\begin{aligned} |A, 3/2\rangle= & {} |\uparrow \uparrow \uparrow \rangle \nonumber \\ |A, 1/2\rangle= & {} \frac{1}{\sqrt{3}}\left( | \uparrow \uparrow \downarrow \rangle + | \downarrow \uparrow \uparrow \rangle + |\uparrow \downarrow \uparrow \rangle \right) \nonumber \\ |E_{+}, 1/2\rangle= & {} \frac{1}{\sqrt{3}}\left( | \uparrow \uparrow \downarrow \rangle + \varepsilon ^{*}| \downarrow \uparrow \uparrow \rangle + \varepsilon |\uparrow \downarrow \uparrow \rangle \right) \nonumber \\ |E_{-}, 1/2\rangle= & {} \frac{1}{\sqrt{3}}\left( | \uparrow \uparrow \downarrow \rangle + \varepsilon | \downarrow \uparrow \uparrow \rangle + \varepsilon ^{*}|\uparrow \downarrow \uparrow \rangle \right) \end{aligned}$$

with $\varepsilon =\hbox {e}^{i \frac{2\pi }{3}}$. The other four eigenstates are obtained by replacing $|\uparrow \rangle$ with $|\downarrow \rangle$. We denote the spin eigenstates with $\{|s, m\rangle \}$ where the first label $s\in \{A, E_{+}, E_{-}\}$ is the “symmetry label” and the second label $m \in \{\pm \frac{3}{2}, \pm \frac{1}{2} \}$ is the collective magnetization. The symmetry label reflects that if we cyclically permute the indistinguishable spins, the $s=A$ eigenstates are invariant and the $s= E_{+}$ ($s=E_{-}$) states acquire a phase $\varepsilon$ (or $\varepsilon ^{*}$). In addition, all $|s, m\rangle$ are eigenstates of the Zeeman Hamiltonian. So, they are labeled by the eigenvalues of the *z* component of the total spin angular momentum operator, $\hbar \ \hat{\mathbf{S }}_{z}=\frac{\hbar }{2} \sum \nolimits _{i=1}^{3} \ \sigma ^{(i)}_{z}$. The above states are the eigenstates of the cyclic permutation operator denoted by $P_{+}$.

As a first approximation, when the chemical shift anisotropy and dipole–dipole interactions are negligible compared to the Zeeman interaction, the Hilbert space of these identical spins can be partitioned as a direct sum of two subspaces, $\mathscr {H}=\mathscr {H}_{A} \oplus \mathscr {H}_{E}$. Each subspace can be further decomposed into a product of the symmetry label *s* and the magnetization label *m*, i.e., $\mathscr {H}_{A}= \mathbb {C}^1 \otimes \mathbb {C}^{4}$ and $\mathscr {H}_{E}=\mathbb {C}^2 \otimes \mathbb {C}^{2}$. The first subsystem (*s*) is not affected by collective noise operators, because

3 $$\begin{aligned} \hat{\mathbf{S }}_{z} \ |s, m \rangle= & {} m\ |s, m\rangle , \nonumber \\ \hat{\mathbf{S }}_{x}\ |s, m \rangle= & {} C_{+}\ |s, m+1 \rangle + C_{-}\ |s, m-1 \rangle , \nonumber \\ \hat{\mathbf{S }}_{y} \ |s, m \rangle= & {} C'_{+}\ |s, m+1 \rangle + C'_{-}\ |s, m-1 \rangle , \end{aligned}$$

where $C_{\pm }$ and $C'_{\pm }$ are complex numbers. All components of the total spin angular momentum, $\hat{\mathbf{S }}_{ \alpha }$ with $\alpha \in \{ x, y, z\}$, preserve the symmetry label although they may corrupt the magnetization label. One can take advantage of this symmetry property by polarizing the methyl group in terms of the symmetry order rather than the conventional Zeeman order. This way, the system is protected against collective spin noise and may exhibit long relaxation times. This has been experimentally demonstrated as *long-lived states* in methyl groups [12] where an imbalance of population is created between the *A* subspace and the $E_{\pm }$ subspaces. In the following section, we provide a mathematical description for these noise- protected states.

We denote a state that is only populated in a particular symmetry subspace by $\rho _{s}$ with $s\in \{A, E_{+}, E_{-}\}$. These states are totally polarized in terms of the symmetry label, but they are totally mixed in terms of the magnetization label. Explicitly, we define these density states by4With the above definitions, we introduce a $\gamma -$polarized long- lived state as

5 $$\begin{aligned} Q_\mathrm{LLS}= & {} \frac{(1+\gamma )}{2}\rho _{A} + \frac{(1-\gamma )}{2} \left( \frac{\rho _{E_{+}}+ \rho _{E_{-}} }{2}\right) \nonumber \\= & {} \frac{ 1}{8}\left( \mathbb {1} + \frac{ \gamma }{3}\ \left( \overrightarrow{\sigma }^{(1)}. \overrightarrow{\sigma }^{(2)} + \overrightarrow{\sigma }^{(2)}. \overrightarrow{\sigma }^{(3)} + \overrightarrow{\sigma }^{(1)}. \overrightarrow{\sigma }^{(3)}\right) \right) . \end{aligned}$$

The appearance of the scalar terms, $\overrightarrow{\sigma }. \overrightarrow{\sigma }$, assures that $Q_\mathrm{LLS}$ is not affected by any component of the total spin magnetization, $\hat{\mathbf{S }}_{\alpha }$, with $\alpha \in \{x,y,z\}$. One could extend this idea and define a more general type of protected states as

6 $$\begin{aligned} Q_{\text {Protected}}=\frac{(1+\gamma )}{2}\rho _{A} + \frac{(1-\gamma )}{2}\left( \frac{1+ \beta }{2} \rho _{E_{+}} + \frac{1- \beta }{2} \rho _{E_{-}} \right) \end{aligned}$$

where the *A* subspace is $\gamma$-polarized relative to the *E* subspace and the $E_{+}$ subspace is $\beta$-polarized relative to the $E_{-}$ subspace. In this report, we are not concerned about the initialization of the system in $Q_\mathrm{LLS}$ or $Q_{\text {Protected}}$. We assume that a long-lived state is given, and we study the dipolar relaxation mechanism that turns this noise-protected state into an NMR observable.

## Symmetry of dipolar coupling

So far, we have neglected that not all interaction terms in the Hamiltonian preserve the cyclic permutation symmetry. Ideally, if the first two terms of Eq. 1 are the only terms in the Hamiltonian, spins are completely indistinguishable, and therefore, the long-lived states can never be converted into an NMR observable. If they are protected against the collective noise, they are protected against the measurement as well. Quantitatively, $\text {Tr}[Q_\mathrm{LLS}\ \mathbf {S}_{\alpha }] = 0$ for all $\alpha \in \{x,y,z\}$. The chemical shift anisotropy, the internal dipolar coupling among protons in a methyl group and the external dipolar coupling between the group of protons and an external spin (such as $^ {13}$C) are all possible interactions that do not preserve the spin symmetry. These physical interactions that distinguish spins can potentially transform the symmetry polarization into Zeeman polarization, leading to NMR observation of what were once protected states. The focus of our study is the contribution of dipole–dipole interaction in the spin read out of an initially prepared protected state. The CSA is normally small for protons in methyl groups in comparison with the dipolar coupling and hence is not considered in this study, although the symmetry arguments would be similar.

### Dipolar Hamiltonian

Two magnetic dipole moments $\mathbf {d}_{1}$ and $\mathbf {d}_{2}$, which are at a distance $\mathbf r$ apart, interact through space via the dipolar Hamiltonian [23],

7 $$\begin{aligned} H_{\text {dip}}= -\, \frac{\mu _{0}}{4 \pi } \frac{1}{ r^{5}}\left( 3(\mathbf {d}_{1}.\mathbf {r})(\mathbf {d}_{2}.\mathbf {r}) - \mathbf {d}_{1}.\mathbf {d}_{2}\right) . \end{aligned}$$

Here, $\mathbf {d}_{1}= \hbar \gamma _{S}\ \mathbf {S}$ and $\mathbf {d}_{2}= \hbar \gamma _{I}\ \mathbf {I}$ with $\mathbf {S}=\frac{1}{2}\overrightarrow{\sigma }$ and $\mathbf {I}=\frac{1}{2}\overrightarrow{\sigma }$ for the case of spin half particles where $\overrightarrow{\sigma }$ are the Pauli operators. The use of different symbols *S* and *I* emphasizes that these operators act on different Hilbert spaces and may represent two different spin species. The dipolar Hamiltonian is commonly written in a product form of spatial functions and irreducible rank-2 tensors [23],

8 $$\begin{aligned} H_{\text {dip}}= & {} \sum \limits _{q=-2}^{2} H_{q} \nonumber \\= & {} c_{0}\ \sum \limits _{q=-2}^{2} \hbox {e}^{-i q\varphi }\ F_{q}( r, \theta ) \ \hat{T}_{q}, \end{aligned}$$

where $c_{0}=-\frac{\hbar \mu _{0} \gamma _{I}\gamma _{S}}{4 \pi }$ is a constant, $F_{q}( r, \theta )$ a function of space parameters and $\hat{T}_{q}$ a normalized bilinear spin operator. Explicitly,Given these definitions, we have

9 $$\begin{aligned} \hat{T}_{q}^{\dagger }= & {} (-1)^{q} \hat{T}_{-q} \nonumber \\ Tr[\hat{T}_{q}^{\dagger } \hat{T}_{q'}]= & {} \delta _{q,q'}, \nonumber \\ F_{-q}= & {} (-1)^{q} F_{q}. \end{aligned}$$

In case of the homonuclear interaction, since $[S_{z}+ I_{z}, \hat{T}_{q}] = q\ \hat{T}_{q}$, each $\hat{T}_{q}$ term changes the *z* component of the total spin magnetization from *m* to $m+ q$. So, we may refer to *q* as the *order* number.

In case of heteronuclear interactions, each $\hat{T}_{q}$ is further decomposed to $\hat{T}_{q} = \sum \nolimits _{p} \ \hat{T}_{(q,p)}$ based on its commutation with the Zeeman Hamiltonian, i.e., $[ \omega _{s} \ S_{z} + \omega _{I}\ I_{z} \ , \ \hat{T}_{(q,p)} ]= \omega _{(q,p)}\ \hat{T}_{(q,p)}$. The explicit form of these bilinear spin operators and their corresponding frequencies are listed in Table 1. One can write them in a closed form by noting $\hat{T}_{(q,p)}\propto S_{p} \otimes I_{q-p}$ and $\omega _{(q,p)}= p\ \omega _{s} + (q-p)\ \omega _{I}$, where $I_{0}$ or $S_{0}$ represents $\frac{\sigma _{z}}{2}$, and $I_{\pm }$ or $S_{\pm }$ represents $\frac{\sigma _{x} \pm i \sigma _{y}}{2}$.

**Table 1:** Components of the dipolar Hamiltonian

*q*	$\hat{T}_{(q,p)}$			$\omega _{(q,p)}$		
	$p=1$	$p=0$	$p=-\,1$	$p=1$	$p=0$	$p=-\,1$
0	$-\,\frac{1}{\sqrt{6}}S_{+}I_{-}$	$\sqrt{\frac{8}{3}} S_{z} I_{z}$	$-\,\frac{1}{\sqrt{6}}S_{-}I_{+}$	$\omega _{s}- \omega _{I}$	0	$-\,\omega _{s}+ \omega _{I}$
+ 1	$S_{+} I_{z}$	$S_{z}I_{+}$	–	$\omega _{s}$	$\omega _{I}$	–
+ 2	$S_{+} I_{+}$	–	–	$\omega _{s}+ \omega _{I}$	–	–

Given the above definitions, the homonuclear coupling between protons in a methyl group is

10 $$\begin{aligned} H^{SS}_{\text {dip}}= & {} H_{\text {dip}}^{(1,2)}+ \ H_{\text {dip}}^{(2,3)}+ H_{\text {dip}}^{(3,1)} \nonumber \\= & {} c_{1}\sum \limits _{i<j} \sum \limits _{q=-2}^{2} \hbox {e}^{-i q\varphi _{ij}}\ F_{q}( r_{ij}, \theta _{ij}) \ \hat{T}^{(i,j)}_{q} \end{aligned}$$

11 $$\begin{aligned}= & {} \sum \limits _{q=-2}^{2} H^{SS}_{q}, \end{aligned}$$

where $H_{\text {dip}}^{(i,j)}$ represents the dipole–dipole coupling between the *i*th proton and the *j*th proton and is given in Eq. 7 with $S= S^{i}$ and $I=S^{j}$. In addition, $\mathbf r _{ij}= (r_{ij}, \theta _{ij}, \varphi _{ij})$ is the relative distance between the two protons and $c_{1}=-\frac{\hbar \mu _{0} \gamma _{I}\gamma _{I}}{4 \pi }$ is a constant.

Similarly, the heteronuclear coupling between the collective spins, $\mathbf {S}$, and the test spin, *I*, consists of three terms,

12 $$\begin{aligned} H^{\mathbf {S}I}_{\text {dip}}= & {} H_{\text {dip}}^{(1)}+ \ H_{\text {dip}}^{(2)}+ H_{\text {dip}}^{(3)} \nonumber \\= & {} c_{0}\sum \limits _{j=1}^{3} \sum \limits _{q,p} \hbox {e}^{-i q\varphi _{j}}\ F_{q}( r_{j}, \theta _{j}) \ \hat{T}^{j}_{(q,p)} \end{aligned}$$

13 $$\begin{aligned}= & {} \sum \limits _{q}\ H^{\mathbf {S}I}_{q}, \end{aligned}$$

where $H_{\text {dip}}^{(j)}$ represents the dipole–dipole coupling between the *j*th proton, $S^{j}$ and the test spin, *I*, and $\mathbf r _{j}= (r_{j}, \theta _{j}, \varphi _{j})$ is the relative distance between them in the Zeeman frame.

The heteronuclear dipolar Hamiltonian does not commute with the spin cyclic permutation operator, i.e., $[P_{+}\ ,\ \sum \nolimits _{q}\ H^{\mathbf {S}I}_{q} ]\ne 0$. Similarly, the homonuclear dipolar Hamiltonian does not commute either. This non-commutativity implies that the dipolar Hamiltonian and the cyclic permutation operator, $\hat{\textit{P}}_{+}$, do not have a common basis, and hence, the dipolar interaction can induce transitions between the eigenstates with different symmetry labels. Consequently, a dipolar relaxation might lead to a process that takes polarization from symmetry order to Zeeman order, which is measurable.

### Symmetrized Operators

We introduce the symmetrized bilinear spin operator and rewrite the dipolar Hamiltonian in terms of them. These symmetrized operators provide insight into the key components of the dipolar coupling that leads to observing a protected state. We start with the external dipolar interaction and then elaborate on the internal dipolar coupling and discuss their differences and similarities.

For heteronuclear coupling, we rewrite the Hamiltonian in Eq. 12 by substituting $B^{j}_{q} = c_{0} \hbox {e}^{-i q \varphi _{j}} \ F_{q}(r_{j}, \theta _{j})$[24, 25] and obtain,

14 $$\begin{aligned} H^{\mathbf {S}I}_{\text {dip}}(t)= & {} \sum \limits _{q,p} \sum \limits _{j=1}^{3}\ B^{j}_{q}(t) \ \hat{T}_{(q,p)}^{j} \nonumber \\= & {} \sum \limits _{q,p}\left\{ + \frac{1}{3} \ \left( B^{1}_{q} + \ B^{2}_{q} + \ B^{3}_{q}\right) \right. \nonumber \\&\times \left( \hat{T}^{1}_{(q,p)}+ \hat{T}^{2}_{(q,p)} + \ \hat{T}^{3}_{(q,p)}\right) \nonumber \\&+\, \frac{1}{3} \ \left( B^{1}_{q} + \varepsilon ^{*} \ B^{2}_{q} + \varepsilon \ B^{3}_{q}\right) \nonumber \\&\times \left( \hat{T}^{1}_{(q,p)} + \varepsilon \ \hat{T}^{2}_{(q,p)} + \varepsilon ^{*}\ \hat{T}^{3}_{(q,p)}\right) \nonumber \\&+\, \frac{1}{3} \ \left( B^{1}_{q} + \varepsilon \ B^{2}_{q} + \varepsilon ^{*} \ B^{3}_{q}\right) \nonumber \\&\left. \times \left( \hat{T}^{1}_{(q,p)} + \varepsilon ^{*}\ \hat{T}^{2}_{(q,p)} + \varepsilon \ \hat{T}^{3}_{(q,p)}\right) \right\} \nonumber \\= & {} \sum \limits _{q,p} \sum \limits _{\lambda =0, \pm 1} \ B_{q}^{\lambda }(t) \ \hat{T}_{(q,p)}^{\lambda }, \end{aligned}$$

where $\varepsilon = \hbox {e}^{\frac{i 2 \pi }{3} }$. We emphasize that in the last line, the sum over different spin indices is replaced by a sum over the symmetry label $\lambda \in \{ 0 , \pm 1\}$, corresponding to $\{A, E_{\pm }\}$ symmetries. The symmetrized complex functions $B^{\lambda }_{q}$ and the symmetrized bilinear spin operator $\hat{T}^{\lambda }_{(q,p)}$ are given by

15 $$\begin{aligned} B^{\lambda }_{q}:= & {} \frac{1}{\sqrt{3}}\ \left( B^{1}_{q} + \varepsilon ^{\lambda *} \ B^{2}_{q} + \varepsilon ^{\lambda } \ B^{3}_{q}\right) , \nonumber \\ \hat{T}^{\lambda }_{(q,p)}:= & {} \frac{1}{\sqrt{3}}\ \left( \hat{T}^{1}_{(q,p)} + \varepsilon ^{\lambda }\ \hat{T}^{2}_{(q,p)} + \varepsilon ^{\lambda *} \ \hat{T}^{3}_{(q,p)}\right) . \end{aligned}$$

In closed form, $\hat{T}^{\lambda }_{(q,p)} \propto \mathbf S _{p}^{\lambda } \otimes I_{q-p}$, where the bold notation reminds us that $\mathbf S _{p}^{\lambda }$ acts on all three spins.

To clarify the effect of $\mathbf S _{p}^{\lambda }$ on the collective spin system, consider a more simple example of two spins, where $\varepsilon = \hbox {e}^{\frac{i 2\pi }{2}}= -1$ and $\lambda \in \{0 ,1\}$ (or $\{A,E\}$). Thus, $\mathbf S _{p}^{A/E} \propto \left( S^{1}_{p} \pm S^{2}_{p} \right)$ is either symmetric or antisymmetric. By looking at the nonzero matrix elements of $\mathbf S _{p}^{\lambda }$ in the triplet–singlet basis, we conclude that the symmetric tensors $\mathbf S ^{A}_{p}$ cause transitions only *within* each symmetry subspace and the antisymmetric tensors $\mathbf S ^{E}_{p}$ cause transitions *between* two subspaces of the triplet states (*A*) and the singlet state (*E*). Indeed, for two spins we obtain

16 $$\begin{aligned} \mathbf S ^{\lambda }_{p}= \frac{1}{\sqrt{2}}\left( S^{1}_{p} + (-1)^{\lambda } S^{2}_{p} \right) \ | j, m\rangle \longrightarrow | j + \lambda , m + p\rangle \end{aligned}$$

where $j=1$ is the triplet subspace and $j=0$ is the singlet subspace.

**Fig. 1:**
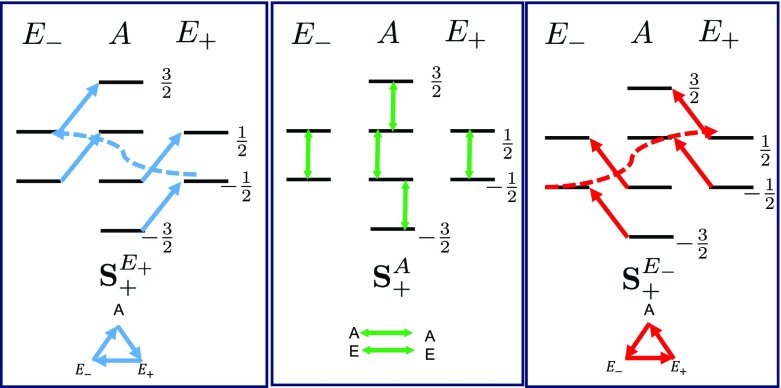
Allowed transitions due to $\mathbf {S}_{+}^{\lambda }$: the nonzero transitions between the eigenstates of $P_{+}$ are shown that are due to the nonzero matrix elements of the symmetrized collective spin operators $\mathbf {S}_{+}^{\lambda }$. The lower index $+$ acts on the magnetization label where it takes *m* to $m+1$ and the upper index $\lambda$ acts on the symmetry label in a cyclic manner. The blue/red arrows indicate the nonzero transitions between different symmetry spaces in the right/left cyclic order, and the green arrows refer to nonzero transition within each symmetry space

Extending this to three spins is straightforward. The $\mathbf S ^{A}_{p}$ of three spins is a totally symmetric operator, and it has nonzero matrix elements only within each symmetry subspaces *A*, $E_{+}$ or $E_{-}$. Consequently, it is block diagonal in the eigenbasis of the cyclic permutation operator. In a similar manner to the two-spin case, $\mathbf S ^{E_{\pm }}_{p}$ connects two different symmetry subspaces. Moreover, in case of three spins, $\mathbf S ^{E_{\pm }}_{p}$ is neither symmetric nor antisymmetric, and one needs to be careful about the direction of transitions. We expand the $\mathbf S ^{E_{\pm }}_{p}$ in the $|s, m\rangle$ basis and conclude that $\mathbf S ^{E_{+}}_{p}$ transforms the symmetry of the eigenstates in the *right cycle* as $(A\rightarrow E_{+}\rightarrow E_{-}\rightarrow A)$ and the $\mathbf S ^{E_{-}}_{p}$ transforms the symmetry of the eigenstates in *left cycle* as $(A\leftarrow E_{+}\leftarrow E_{-}\leftarrow A)$. The upper index of $\mathbf S ^{\lambda }_{p}$ determines whether the transformation is *within* each symmetry subspace $(\lambda =A)$ or *between* them in a right/left cyclic direction ($\lambda = E_{\pm })$. The lower index determines the change in the magnetization. Explicitly, when $p=0$, $\mathbf S ^{\lambda }_{0}$ takes $m\rightarrow m$ and when $p=\pm 1$ the $\mathbf S ^{\lambda }_{\pm }$ takes $m\rightarrow m \pm 1$ for all values of $\lambda$. In other words,

17 $$\begin{aligned} \mathbf S ^{\lambda }_{p}\ | s, m\rangle \longrightarrow | s + \lambda , m + p\rangle \end{aligned}$$

where the sum in $s + \lambda$ is mod 3. The final remark is that the reverse transition process occurs via $(\mathbf S ^{\lambda }_{p})^{\dagger }$, where

18 $$\begin{aligned} \left( \mathbf {S}_{p}^{\lambda }\right) ^{\dagger } = \mathbf {S}_{-p}^{-\lambda }. \end{aligned}$$

For example, the transition from $|E_{+}, \frac{1}{2}\rangle$ to $|A, \frac{3}{2}\rangle$ occurs through $\mathbf {S}_{+}^{E_{+}}$ but the reverse process, from $|A, \frac{3}{2}\rangle$ to $|E_{+}, \frac{1}{2}\rangle$ occurs through $\mathbf {S}_{-}^{E_{-}}$. The allowed transitions due to $\mathbf {S}_{+}^{\lambda }$ are demonstrated in Fig. 1.

The homonuclear dipolar Hamiltonian can also be written as a sum over symmetrized operators. We substitute $G^{ij}_{q} = c_{1} \hbox {e}^{-i q \varphi _{ij}} \ F_{q}(r_{ij}, \theta _{ij})$ in Eq. 10 and rewrite the Hamiltonian as,

19 $$\begin{aligned} H^{SS}_{\text {dip}}(t) =\sum \limits _{q,p}&\sum \limits _{i<j}^{3}\ G^{ij}_{q}(t) \ \hat{T}_{q}^{(i,j)} \nonumber \\ = \sum \limits _{q,p}&\sum \limits _{\lambda =0, \pm 1} \ G_{q}^{\lambda }(t) \ \hat{\mathbb {T}}_{(q,p)}^{\lambda }, \end{aligned}$$

where

20 $$\begin{aligned} G^{\lambda }_{q}:= & {} \frac{1}{\sqrt{3}}\ \left( G^{12}_{q} + \varepsilon ^{\lambda *} \ G^{23}_{q} + \varepsilon ^{\lambda } \ G^{31}_{q}\right) , \nonumber \\ \hat{\mathbb {T}}^{\lambda }_{q}:= & {} \frac{1}{\sqrt{3}}\ \left( \hat{T}^{(1,2)}_{q} + \varepsilon ^{\lambda }\ \hat{T}^{(2,3)}_{q} + \varepsilon ^{\lambda *} \ \hat{T}^{(3,4)}_{q}\right) . \end{aligned}$$

**Fig. 2:**
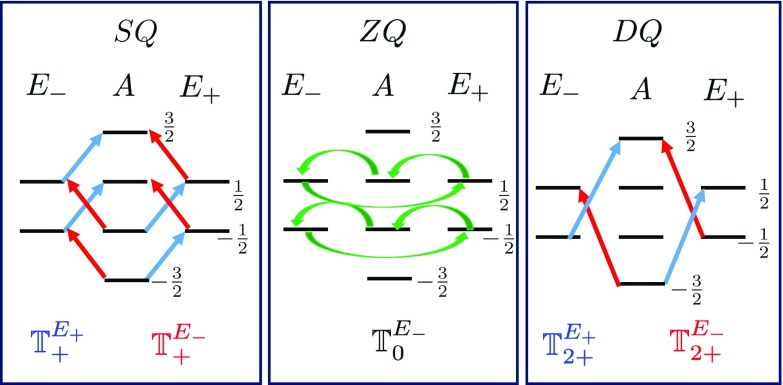
Allowed transitions due to $\mathbb {T}_{q}^{\lambda }$: the nonzero transitions between the eigenstates of $P_{+}$ are shown that are due to the nonzero matrix elements of the symmetrized bilinear spin operators $\mathbb {T}_{q}^{\lambda }$. From left to right, the figure shows allowed transitions due to the SQ ($q=1$), the ZQ ($q=0$) and the DQ ($q=2$) spin operators. The blue/red arrows indicate the nonzero transitions between different symmetry spaces in the right/left cyclic. The green arrows indicate those transitions that do not effectively contribute in the NMR signal

We observe that the symmetrized spin operators for internal dipolar coupling, $\mathbb {T}_{q}^{\lambda }$, exhibit similar symmetry properties to that of the external dipolar coupling, $\mathbf {S}_{p}^{\lambda }$. Similarly, the fully symmetrized operators, $\lambda =0$ or *A*, have nonzero matrix elements only *within* each symmetry subspace and not between them, and hence, they are not of interest. Thus, the important terms are $\lambda = E_{\pm }$, and Fig. 2 shows all the nonzero transitions induced by $\mathbb {T}_{q}^{\lambda }$ for $q=0,1,2$. Note that in the figure the $S_{z}^{i} S^{j}_{z}$ terms are not considered because they contribute to shifting the energy levels not to the transitions.

The above transitions can be categorized in three groups: the zero-quantum transitions (ZQ) also known as * flip–flop* terms when $q=0$ and $m \rightarrow m$, the single-quantum transitions (SQ) or *single spin flip* terms when $q=1$ and $m \rightarrow m \pm 1$, and the double-quantum transitions (DQ) or the *flop–flop* terms when $q=2$ and $m \rightarrow m \pm 2$. Similar to the $\mathbf {S}_{\pm }^{\lambda }$ operators in case of heteronuclear Hamiltonian, the internal flip–flop interaction among protons also leads to transitions between states with different symmetry in a cyclic order (left /right for $\lambda =E_{\mp }$). More precisely,

21 $$\begin{aligned} \hat{ \mathbb {T}}_{0}^{\lambda } \ | s\, \ m\rangle \rightarrow |s+ \lambda \ ,\ m\rangle (A\rightleftarrows E_{+}\rightleftarrows E_{-}\rightleftarrows A) \end{aligned}$$

These transitions are *passive* in the sense that even though they lead to an exchange of population between different symmetry subspaces, they will not effectively contribute in transferring polarization from the symmetry order to Zeeman order, as it is explained in the next section.

The $\text {SQ}$ and the $\text {DQ}$ transitions, the $\hat{ \mathbb {T}}_{+1}^{\lambda }$ and the $\hat{ \mathbb {T}}_{+2}^{\lambda }$ operators, are slightly different. They do induce transitions between the *A* and the $E_{\pm }$ subspaces but do not allow transition between the $E_{+}$ and the $E_{-}$ subspaces as opposed to the external dipolar coupling. For $q= 1 ,2$ we have

$$\begin{aligned}&\hat{\mathbb {T}}_{q}^{E_{+}}: | A\ ,\ m\rangle \rightleftarrows |E_{\pm } \ , \ m +q \rangle A\rightleftarrows E_{\pm } \\&\hat{\mathbb {T}}_{q}^{E_{+}}: | A\ ,\ m\rangle \rightleftarrows |E_{\pm } \ , \ m +q \rangle A\rightleftarrows E_{\pm } \end{aligned}$$

Now that we visualized the effect of these symmetrized irreducible tensors on the symmetrized eigenbasis, and we proceed to solve the master equation in the next section.

## Master equation

At high temperature and in liquid phase, the space coordinates $\mathbf r _{j}(t)= (r_j, \theta _{i}, \varphi _{i})$ randomly fluctuate in time, as does the dipolar Hamiltonian. In the absence of CSA, the total Hamiltonian consists of a Zeeman term $H_{0} = \omega _{h} \ \hat{\mathbf {S}}_{z} + \omega _{I} \ \hat{I}_{z}$, a scalar coupling $H_{\text {scalar}}= 2\pi \ (J_{0} \ \sum \nolimits _{ij} \overrightarrow{\sigma }^{i}. \overrightarrow{\sigma }^{j} + J_{1} \ \mathbf {S}_{z}\ I_{z})$, and a fluctuating dipolar term $H^{\mathbf {S}I}_{\text {dip}}(t)$ and $H^{SS}_{\text {dip}}(t)$. The scalar coupling is totally symmetric and does not contribute to breaking the symmetry of the protected states, so, it is neglected in the following discussion, unless otherwise stated. In the following discussion, we analyze one of the two types of dipolar relaxation process (homo vs. hetero) in the absence of the other. Of course, in experiment, these two processes are not independent of each other and the evolution of one affects the other, because their corresponding Hamiltonians do not commute. For a short time period, $\delta t$, one may consider them as two independent processes. We first analyze the pure contribution of heteronuclear coupling in the NMR observation of initially prepared protected states and then analyze the contribution of the homonuclear coupling and discuss their differences and similarities.

### Relaxation induced by heteronuclear coupling

At high field, we treat $H^{\mathbf {S}I}_{\text {dip}}(t)$ as a perturbed term and apply Redfield’s semiclassical theory [23, 26] to study the collective spin dynamics. Then, we solve the master equation analytically and predict the NMR signal.

The Lindbladian form of the semiclassical master equation gives us [26]

$$\begin{aligned} \frac{\partial \tilde{\rho }}{\partial t} = \sum \limits _{\lambda } \sum \limits _{q,p} J^{\lambda }_{q}\left( \omega _{(q,p)}\right) \hat{T}_{(q,p)}^{\lambda }\ \tilde{\rho }\ \hat{T}_{(q,p)}^{\lambda \dagger } - \frac{1}{2}\left\{ \hat{T}_{(q,p)}^{\lambda \dagger }\hat{T}_{(q,p)}^{\lambda },\ \tilde{\rho }\right\} , \end{aligned}$$

where $\tilde{\rho }= \hbox {e}^{iH_{0} t} \rho \hbox {e}^{-iH_{0}t}$ is the density matrix in the rotating frame of the Zeeman interaction. The coefficient $J^{\lambda }_{q}(\omega )$ is the real part of the symmetrized spectral density of noise and is defined as:

22 $$\begin{aligned} J^{\lambda }_{q}(\omega ):= & {} \int \limits _{-\infty }^{\infty } R_{q}^{\lambda }(\tau ) \hbox {e}^{-i \omega \tau } \hbox {d}\tau , \nonumber \\ R_{q}^{\lambda }(\tau ):= & {} \overline{ B^{\lambda }_{q}(t) (B^{\lambda }_{q})^{*}(t+\tau )}. \end{aligned}$$

Here, $R_{q}^{\lambda }(\tau )$ denotes the symmetrized autocorrelation function, and the overbar notation refers to averaging over the random variables. The imaginary part of the spectral density of noise leads into the dynamical shift and can be absorbed in the coherence evolution part [27].

*For the purpose of the following discussion, the explicit form of the* $J(\omega )$ *is not required, because we are not interested in the exact dynamics of the system. Rather we would like to know which components of the heteronuclear dipolar coupling convert the symmetry polarization of the* $Q_\mathrm{LLS}$ *into the measurable Zeeman polarization.*

We define the *Lindbladian map* with $\hat{D}[L][.]:= \hat{L}\ .\ \hat{L}^{\dagger } - \frac{1}{2}\{ \hat{L}^{\dagger }\hat{L},\ .\}$ in which *L* is the Lindblad operator and rewrite the master equation as

23 $$\begin{aligned} \frac{\partial \tilde{\rho }}{\partial t} = \sum \limits _{\lambda } \sum \limits _{q,p} J^{\lambda }_{q}(\omega _{(q,p)})\ \eta _{q,p} \ \hat{D}[\mathbf S _{p}^{\lambda } \otimes I_{q-p}][\ \tilde{\rho }]. \end{aligned}$$

where $\eta _{0.0}=8/3$, $\eta _{0,\pm } =-1/6$ and $\eta _{q,p}=1$ for all $q\ne 0$ cases. Considering the classical noise $J^{\lambda }_{q}(\omega ) = J^{-\lambda }_{-q}(-\omega )$ and neglecting the $q=p=0$ term which just shifts the energy, one can break the above master equation into three parts: the zero-quantum transitions (ZQ), the double-quantum transitions (DQ) and the single-quantum transitions (SQ),

24 $$\begin{aligned} \frac{\partial \tilde{\rho }}{\partial t}= & {} \sum \limits _{\lambda } \text {ZQ}^{\lambda }[\tilde{\rho }] + \text {DQ}^{\lambda }[\tilde{\rho }] + \text {SQ}{\lambda }[\tilde{\rho }] \nonumber \\ \text {ZQ}^{\lambda }[.]:= & {} \frac{1}{6}\ J^{\lambda }_{0}(\omega _{s}- \omega _{I})\ \left( \hat{D}[ \mathbf S _{+}^{\lambda } \otimes I_{-}][.] + \hat{D}[ \mathbf S _{-}^{-\lambda } \otimes I_{+}][.] \right) \nonumber \\ \text {DQ}^{\lambda }[.]:= & {} J^{\lambda }_{2}(\omega _{s} +\omega _{I})\ \left( \hat{D}[ \mathbf S _{+}^{\lambda } \otimes I_{+}][.] + \hat{D}[ \mathbf S _{-}^{-\lambda } \otimes I_{-}][.] \right) \nonumber \\ \text {SQ}{\lambda }[.]:= & {} J^{\lambda }_{1}( \omega _{s}) \ \left( \hat{D}[ \mathbf S _{+}^{\lambda } \otimes I_{z}][.] + \hat{D}[ \mathbf S _{-}^{-\lambda } \otimes I_{z}][.] \right) \nonumber \\&+\,J^{\lambda }_{1}( \omega _{I}) \ \left( \hat{D}[ \mathbf S _{z}^{\lambda } \otimes I_{+}][.] + \hat{D}[ \mathbf S _{z}^{-\lambda } \otimes I_{-}][.] \right) . \end{aligned}$$

Here, we replace $(\mathbf {S}_{p}^{\lambda })^{\dagger } = \mathbf {S}_{-p}^{-\lambda }$. The ZQ and the DQ terms exchange energy between the collective spin and the test spin, and the SQ terms change either the collective spin states or the test spin states. As was mentioned before, the totally symmetric Lindblad operators $\mathbf S _{p}^{A} \otimes I_{q-p}$ do not cause a transition between the two different symmetry subspaces of the collective spin. Therefore, if it happens that the spectral density of noise is very well approximated with just the totally symmetric component, i.e., $J^{\lambda }_{q}(\omega ) \approx J^{A}_{q}(\omega )$, one can conclude that the system is very robust against noise and exhibits very long relaxation time. This is in agreement with the result in [12, 17] where in the limit of very fast rotations of the methyl group, the LLS becomes the eigenstate of the relaxation superoperator, preserving the population imbalance. For the sake of simplicity in the following discussion, we ignore all of the totally symmetric Lindblad operators, since they do not play a critical role in observing the protected state. We also neglect the $\text {SQ}^{\lambda }[.]$ terms, because we are interested in those transitions that the collective spin exchanges the energy with the test spin. The only important terms in the dissipator are $\text {DQ}^{E_{\pm }}$ and $\text {ZQ}^{E_{\pm }}$.

To obtain the allowed transitions due to ZQ and DQ terms, we need to calculate the effect of $\hat{D}[ \mathbf S _{p}^{\lambda } \otimes I_{\pm }] [.]$ on the eigenbasis $\{ |s, m\rangle \otimes |\uparrow \rangle \text {or} |\downarrow \rangle \}$. The nonzero components are,

25 $$\begin{aligned} \hat{D}[ \mathbf S _{p}^{\lambda } \otimes I_{-}][|s,m\rangle \langle s,m| \otimes | \uparrow \rangle \langle \uparrow | ]= & {} \mathbf S _{p}^{\lambda }\ |s,m\rangle \langle s,m| \ \mathbf S _{p}^{\lambda })^{\dagger } \otimes I_{-}\ | \uparrow \rangle \langle \uparrow |\ I_{+} \nonumber \\&-\, \frac{1}{2}\left( \mathbf S _{-p}^{-\lambda }{} \mathbf S _{p}^{\lambda } \ |s,m\rangle \langle s,m|\ \otimes I_{+}I_{-} \ | \uparrow \rangle \langle \uparrow | \right) \nonumber \\&-\, \frac{1}{2}\left( |s,m\rangle \langle s,m| \ \mathbf S _{-p}^{-\lambda }{} \mathbf S _{p}^{\lambda } \otimes | \uparrow \rangle \langle \uparrow |\ I_{+}I_{-} \right) \nonumber \\\propto & {} |s+\lambda , m+p \rangle \langle s+\lambda , m+p| \otimes | \downarrow \rangle \langle \downarrow | \nonumber \\&-\, |s, m\rangle \langle s, m| \otimes | \uparrow \rangle \langle \uparrow |. \end{aligned}$$

Similarly,

$$\begin{aligned}&\hat{D}[ \mathbf S _{p}^{\lambda } \otimes I_{+}]\left[ |s,m\rangle \langle s,m| \otimes | \downarrow \rangle \langle \downarrow | \right] \propto |s+\lambda , m+p \rangle \\&\qquad \langle s+\lambda , m +p| \otimes | \uparrow \rangle \langle \uparrow |- |s, m\rangle \langle s, m| \otimes | \downarrow \rangle \langle \downarrow |. \end{aligned}$$

The proportionality constant is 1 for transitions from or to the $m=\pm 3/2$ and is $\frac{1}{3}$ for all other levels.

**Fig. 3:**
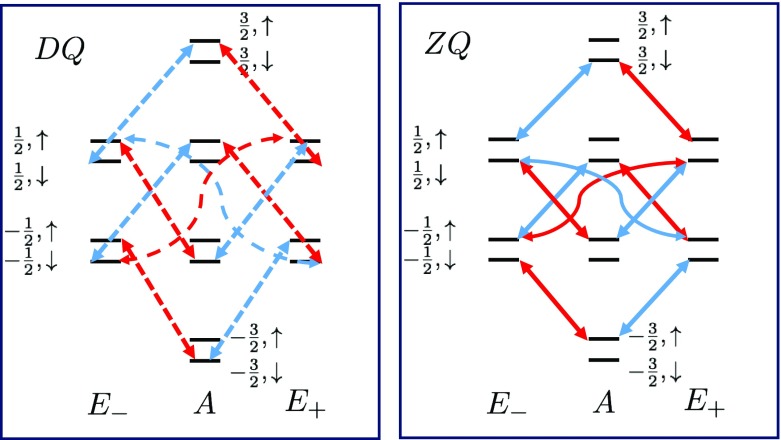
Left segment: the selection rule due to $\text {DQ}^{E_{\pm }}$ transitions. Right segment: the selection rule due to $\text {ZQ}^{E_{\pm }}$ transitions. Red versus blue refers to nonzero transitions due to $\mathbf {S}^{E_{-}}_{p}$ versus $\mathbf {S}^{E_{+}}_{p}$

Based on the above relations, the allowed transition due to $\text {ZQ}^{E_{\pm }}$ and $\text {DQ}^{E_{\pm }}$ terms is indicated in Fig. 3 in which $\lambda = E_{\pm }$ transitions are color coded with red and blue, respectively.

The calculation in Eq. 25 convinces us that if the initial state is a probabilistic mixture of different energy levels, the above master equation reduces to the classical rate equations to:

26 $$\begin{aligned} \frac{\mathrm{d}}{\mathrm{d}t}[x](t) = \sum \limits _{y\ne x} W_{xy}\ \left( [y](t)- [x](t)\right) , \end{aligned}$$

where [*x*](*t*) is the population of the *x*th energy level at time *t* with $x \in \{ |s,m\rangle \otimes ( | \uparrow \rangle \text {or} | \downarrow \rangle ) \}$, and $W_{xy}$ is the transition rate between two energy levels *x* and *y*. To solve the above differential equation, the evolution time is discretized into *N* steps where $t_{N}= N\delta t$ and $\delta t$ is small compared to the energy scales of the system. Given the population distribution at time $t_{n}$, the change in the population of *x*th level at time $t_{n+1}$ is approximated by $\varDelta [x](n+1)\approx \delta t \times \sum \nolimits _{xy} \ W_{xy}\ \left( [y](n) - [x](n)\right)$. Therefore, if the initial conditions are known, one should be able to calculate the NMR signal by solving the rate equations recursively. Note that at any instance of time, $t= t_{0}+ \delta t$ the transition occurs only between those energy levels that first are allowed due to $\text {ZQ}^{E_{\pm }}$ and $\text {DQ}^{E_{\pm }}$ terms, and second, there is an imbalance of population at $t=t_{0}$.

The initial state of interest is27where $\alpha$ is the polarization of the test spin and $Q_{\text {Protected}}$ is replaced from Eq. 6. Since $\rho _{0}$ is diagonal, we alternatively represent it by a *population vector*
28where29The $\mathbf {q}_{0}$ represents the vector of population of three identical spins at $t=0$ in which all energy levels with the same symmetry label are equally populated, but there is an imbalance of population between energy levels with different symmetry labels. For use in future analysis, we compute

30 $$\begin{aligned} C_{\pm }:= & {} + \frac{\gamma }{4}\mp \beta \frac{(1-\gamma )}{8}, \nonumber \\ C_{2}:= & {} \beta \frac{(1-\gamma )}{4}= C_{-}-C_{+}, \end{aligned}$$

where $C_{\pm }$ is the imbalance of population between the $|A, m\rangle$ states and $|E_{\pm }, m'\rangle$ states and $C_{2}$ is the imbalance of population between $|E_{+} , m'\rangle$ states and $|E_{-} , m'\rangle$ states for all possible values of *m* and $m'$ and at $t=0$.

Considering an unpolarized test spin, $\alpha =0$, the solutions to the rate equations for short evolution time, $t=\delta t$, are

31 $$\begin{aligned} \varDelta \left[ A, \frac{3}{2}, \uparrow \right] _{1}= & {} -\delta t \ \left( C_{+} \ J_{2}^{E_{-}} + C_{-}\ J_{2}^{E_{+}}\right) , \nonumber \\ \varDelta \left[ A, \frac{3}{2}, \downarrow \right] _{1}= & {} -\frac{1}{6}\delta t \ \left( C_{+} \ J_{0}^{E_{-}} + C_{-}\ J_{0}^{E_{+}}\right) , \nonumber \\ \varDelta \left[ A, -\frac{3}{2}, \uparrow \right] _{1}= & {} -\frac{1}{6}\delta t \ \left( C_{+} \ J_{0}^{E_{+}} + C_{-}\ J_{0}^{E_{-}}\right) , \nonumber \\ \varDelta \left[ A, -\frac{3}{2}, \downarrow \right] _{1}= & {} -\delta t \ \left( C_{+} \ J_{2}^{E_{+}} + C_{-}\ J_{2}^{E_{-}}\right) ,\nonumber \\ \varDelta \left[ E_{\pm }, \frac{1}{2}, \uparrow \right] _{1}= & {} \delta t \ \left( \frac{1}{3}\left( C_{\pm } \ J_{2}^{E_{\pm }} \mp C_{2}\ J_{2}^{E_{\mp }}\right) + \frac{1}{ 6}C_{\pm } \ J_{0}^{E_{\mp }}\right) , \nonumber \\ \varDelta \left[ E_{\pm }, \frac{1}{2}, \downarrow \right] _{1}= & {} \delta t \ \left( \frac{1}{3 \times 6} \left( C_{\pm } \ J_{0}^{E_{\pm }} \mp C_{2}\ J_{0}^{E_{\mp }}\right) + C_{\pm } \ J_{2}^{E_{\mp }}\right) , \nonumber \\ \varDelta \left[ E_{\pm }, -\frac{1}{2}, \uparrow \right] _{1}= & {} \delta t \ \left( \frac{1}{3 \times 6} \left( C_{\pm } \ J_{0}^{E_{\mp }} \mp C_{2}\ J_{0}^{E_{\pm }}\right) + C_{\pm } \ J_{2}^{E_{\pm }}\right) , \nonumber \\ \varDelta \left[ E_{\pm }, -\frac{1}{2}, \downarrow \right] _{1}= & {} \delta t \ \left( \frac{1}{3} \left( C_{\pm } \ J_{2}^{E_{\mp }} \mp C_{2}\ J_{2}^{E_{\pm }}\right) + \frac{1}{ 6} C_{\pm } \ J_{0}^{E_{\pm }}\right) . \end{aligned}$$

where we denoted $J^{\lambda }_{2}(\omega _{s}+\omega _{I})$ with $J^{\lambda }_{2}$ and $J^{\lambda }_{0}(\omega _{s}-\omega _{I})$ with $J^{\lambda }_{0}$ for short. Since all energy levels in *A* subspace are equally populated at $t=0$, in the absence of $\text {DQ}^{A}$ and $\text {ZQ}^{A}$ terms, we obtain

$$\begin{aligned} \varDelta \left[ A, \pm \frac{1}{2},\uparrow \right] _{1}= & {} \frac{1}{3} \varDelta \left[ A, \pm \frac{3}{2} ,\uparrow \right] _{1},\\ \varDelta \left[ A, \pm \frac{1}{2} , \downarrow \right] _{1}= & {} \frac{1}{3} \varDelta \left[ A, \pm \frac{3}{2} , \downarrow \right] _{1}. \end{aligned}$$

The expressions in Eq. 31 may appear very complicated and may sound difficult to get an insight into the relaxation. If we pay attention to the symmetry, there is a delicate and simple relation between the population of different energy levels. For all values of (*s*, *m*), the change in the population of $|s, m, \uparrow \rangle$ level is the same as that of $|s, m, \downarrow \rangle$ with just the difference of replacing $J^{\lambda }_{2} \leftrightarrow J^{\lambda }_{0}/6$. Second, the change of population in each level $|s, m, \uparrow \rangle$ is the same as that of $|s, -m, \uparrow \rangle$ with just the difference of replacing $J^{\lambda }_{2} \leftrightarrow J^{-\lambda }_{0}/6$. It will be shown that the anti-phase feature of the NMR peaks observed by others arises from these two properties, which are also visually captured from Fig. 3.

For the $m=\pm \frac{1}{2}$ subspace, after summing over the symmetry labels and doing some algebra, we obtain

32 $$\begin{aligned} \sum \limits _{s} \varDelta \left[ s,\pm \frac{1}{2} ,\uparrow \right] _{1}= & {} - \varDelta \left[ A, \pm \frac{3}{2}, \downarrow \right] _{1}, \nonumber \\ \sum \limits _{s} \varDelta \left[ s,\pm \frac{1}{2} ,\downarrow \right] _{1}= & {} - \varDelta \left[ A, \pm \frac{3}{2}, \uparrow \right] _{1}, \end{aligned}$$

where $s\in \{A, E_{\pm }\}$. We see in the following that these very neat relations between different energy levels enable us to anticipate the NMR signal analytically.

To be more specific, we start from the NMR signal on the test spin channel. The scalar coupling $2\pi \ J_{1}\ \mathbf {S}_{z}\ I_{z}$ that was neglected so far shifts the frequency of the test spin condition on the total spin magnetization of protons. Therefore, it is expected to observe four distinguishable peaks on the *I* channel corresponding to $m=\pm \frac{3}{2}$ and $m=\pm \frac{1}{2}$. We define a set of operators that project the collective spins into these magnetization subspaces with:

33 $$\begin{aligned} \varPi ^{\pm \frac{3}{2}}= & {} \bigg | A, \pm \frac{3}{2} \bigg \rangle \bigg \langle A, \pm \frac{3}{2}\bigg |, \nonumber \\ \varPi ^{\pm \frac{1}{2}}= & {} \sum \limits _{s} \bigg | s, \pm \frac{1}{2} \bigg \rangle \bigg \langle s, \pm \frac{1}{2}\bigg |. \end{aligned}$$

For a short evolution time, the expected NMR peaks at the test spin channel are

$$\begin{aligned} \langle \varPi ^{\pm \frac{3}{2}} \otimes I_{z} \rangle \vert _{\alpha =0}^{\delta t}= & {} \frac{1}{2}\left( \left[ A,\pm \frac{3}{2} ,\uparrow \right] _{\delta t} - \left[ A,\pm \frac{3}{2} ,\downarrow \right] _{\delta t} \right) \\= & {} \frac{\delta t}{8}\ \Big [ \pm \gamma \ \left( \varGamma ^{E_{-}} +\varGamma ^{E_{+}}\right) - \,\beta \frac{(1-\gamma )}{2}\ \left( \varGamma ^{E_{+}} - \varGamma ^{E_{-}}\right) \Big ], \\ \langle \varPi ^{\pm \frac{1}{2}} \otimes I_{z} \rangle \vert _{\alpha =0}^{\delta t}= & {} \frac{1}{2}\left( \sum \limits _{s} \left[ s,\pm \frac{1}{2} ,\uparrow \right] _{\delta t} - \left[ s,\pm \frac{1}{2} ,\downarrow \right] _{\delta t} \right) \\= & {} \langle \varPi ^{\pm \frac{3}{2}} \otimes I_{z} \rangle \vert _{\alpha =0}^{\delta t}, \end{aligned}$$

in which $\varGamma ^{\lambda }:= J_{2}^{\lambda } - \frac{1}{6} J_{0}^{\lambda }$. To compute the above NMR signals, we used $[x]_{\delta t} = [x]_{0} + \varDelta [x]_{1}$ and replaced the expressions from Eq. 31 into it. On the carbon channel, both $\mathbf {S}_{z} \otimes I_{z}$ and $\mathbb {1} \otimes I_{z}$ parts of the density matrix become an NMR observable, leading to an anti-phase term that is proportional to $\gamma$ and an in-phase term that is proportional to $\beta$. Similarly, the anticipated NMR peaks on the proton channel are

$$\begin{aligned} \langle \mathbf {S}_{z} \otimes |\uparrow \rangle \langle \uparrow | \rangle \vert _{\alpha =0}^{\delta t}= & {} \frac{3}{2}\ \left( \left[ A, \frac{3}{2}, \uparrow \right] _{\delta t} - \left[ A, -\frac{3}{2},\uparrow \right] _{\delta t} \right) \\&\quad + \frac{1}{2} \sum \limits _{s} \left( \left[ s, \frac{1}{2}, \uparrow \right] _{\delta t} - \left[ s, -\frac{1}{2},\uparrow \right] _{\delta t} \right) \\= & {} \frac{\delta t}{8}\ \Big [ \gamma \ \left( \tilde{\varGamma }^{E_{+}} +\tilde{\varGamma }^{E_{-}}\right) \\&\quad + \beta \frac{(1-\gamma )}{2}\ \left( \tilde{\varGamma }^{E_{+}} - \tilde{\varGamma }^{E_{-}}\right) \Big ] \\= & {} - \langle \mathbf {S}_{z} \otimes |\downarrow \rangle \langle \downarrow | \rangle \vert _{\alpha =0}^{\delta t} \end{aligned}$$

in which $\tilde{\varGamma }^{\lambda }:= J_{2}^{\lambda } - \frac{1}{6} J_{0}^{-\lambda }$. On the proton channel, $\mathbf {S}_{z} \otimes I_{z}$ becomes an NMR observable, leading to two equal peaks with opposite phases.

It remains to solve the rate equations in case of $\alpha \ne 0$. When the test spin has some initial polarization, $\alpha \ne 0$, in Eq. 30 additional terms show up. Precisely, the imbalance of population difference between different energy levels is now replaced by

34 $$\begin{aligned} \big [A,m, \uparrow \!\big ]_{0}-\left[ E_{\pm },m',\downarrow \right] _{0}= & {} C_{\pm } + \left( \frac{\alpha }{4} \pm \alpha \beta \frac{(1-\gamma )}{8}\right) , \nonumber \\ \big [A, m, \downarrow \!\big ]_{0}-\left[ E_{\pm }, m',\uparrow \right] _{0}= & {} C_{\pm } - \left( \frac{\alpha }{4} \pm \alpha \beta \frac{(1-\gamma )}{8}\right) , \nonumber \\ \left[ E_{+},m', \uparrow \right] _{0}-\left[ E_{-}, m',\downarrow \right] _{0}= & {} C_{2} + \alpha \frac{\ (1-\gamma )}{4}, \nonumber \\ \left[ E_{+},m',\downarrow \right] _{0}-\left[ E_{-},m',\uparrow \right] _{0}= & {} C_{2} - \alpha \frac{\ (1-\gamma )}{4}, \end{aligned}$$

$\forall m\in \{\pm \frac{3}{2}, \pm \frac{1}{2}\}$ and $\forall m'\in \{\pm \frac{1}{2}\}$. By replacing these initial imbalance of population in the rate equation (Eq. 26) and after doing some tedious calculations, we obtain

35 $$\begin{aligned}&\langle \varPi ^{\pm \frac{3}{2}} \otimes I_{z} \rangle \vert _{\alpha \ne 0}^{\delta t} - \langle \varPi ^{\pm \frac{3}{2}} \otimes I_{z} \rangle \vert _{\alpha = 0}^{\delta t} \nonumber \\&\quad =\frac{\delta t}{8}\left[ \alpha \ \left( \tilde{\varGamma }^{E_{+} }+ \tilde{\varGamma }^{E_{-}}\right) \pm \alpha \beta \frac{(1-\gamma )}{2} \ \left( \tilde{\varGamma }^{E_{+}} - \tilde{\varGamma }^{E_{-}}\right) \right] \nonumber \\&\langle \varPi ^{\pm \frac{1}{2}} \otimes I_{z} \rangle \vert _{\alpha \ne 0}^{\delta t} - \langle \varPi ^{\pm \frac{1}{2}} \otimes I_{z} \rangle \vert _{\alpha =0}^{\delta t} \nonumber \\&\quad =\frac{\delta t}{8} \left[ \alpha \ \left( \tilde{\varGamma }^{E_{+} }+ \tilde{\varGamma }^{E_{-}}\right) \pm \alpha \frac{(2\beta -1)}{3} \frac{(1-\gamma )}{2} \ \left( \tilde{\varGamma }^{E_{+}} - \tilde{\varGamma }^{E_{-}}\right) \right] \end{aligned}$$

Interestingly, all terms that have $\beta$ dependency are proportional to $J^{E_{+}}(\omega ) - J^{E_{-}}(\omega )$. This means that the polarization difference between the $E_{+}$ and the $E_{-}$ subspaces become observable if $J^{E_{+}}(\omega ) \ne J^{E_{-}}(\omega )$. This also means that if one is able to create an imbalance of population between the $E_{+}$ and $E_{-}$ subspaces, that wavefunction leads to an extra feature in the observed NMR spectra which reveals information about the symmetry of the spectral density of noise.

In case of $J^{E_{+}}(\omega )= J^{E_{-}}(\omega )$, Eq. 35 reduces to

$$\begin{aligned} \langle \varPi ^{\pm \frac{3}{2}} \otimes I_{z} \rangle \vert _{\alpha \ne 0}^{\delta t}= & {} \frac{\delta t}{8}\ \left[ \pm \gamma \ \left( \varGamma ^{E_{-}} +\varGamma ^{E_{+}}\right) - \alpha \ \left( \tilde{\varGamma }^{E_{+} }+ \tilde{\varGamma }^{E_{-}}\right) \right] \\ \langle \varPi ^{\pm \frac{1}{2}} \otimes I_{z} \rangle \vert _{\alpha \ne 0}^{\delta t}= & {} \frac{\delta t}{8}\ \left[ \pm \gamma \ \left( \varGamma ^{E_{-}} +\varGamma ^{E_{+}}\right) - \alpha \ \left( \tilde{\varGamma }^{E_{+} }+ \tilde{\varGamma }^{E_{-}}\right) \right] \end{aligned}$$

The anti-phase contribution in the NMR spectra is proportional to $\gamma$, the initial imbalance of population between the *A* and *E* symmetry subspaces. The in-phase contribution is proportional to $\alpha$, which is the polarization of the test spin that is dipolarly coupled with the three protons. We do not know the explicit value of different orders of the spectral density of noise and therefore cannot make any conclusion about the relative magnitude of these two contributions. Nevertheless, we can conclude that the peaks in pair of $(\frac{3}{2},\frac{1}{2})$ or $(-\frac{3}{2}, -\frac{1}{2})$ have the same phase and the same amplitude. Now, if $J^{E_{+}}(\omega )\ne J^{E_{-}}(\omega )$, even for the case of $\beta =0$, we can no longer make any judgment about the relative amplitude and phase of these four peaks. Because, according to Eq. 35, even when $\beta =0$ an additional anti-phase term survives for the $m=\pm \frac{1}{2}$ peaks. In “Appendix,” we compute the NMR peaks for one step further when $t=2 \delta t$ and conclude that for a longer evolution time, additional terms show up that differentiates the amplitude of the $m=\pm \frac{3}{2}$ from that of $m=\pm \frac{1}{2}$. Thus, four peaks with unequal amplitudes are expected. This argument holds true without any assumptions about the magnitude of the anti-phase contribution relative to the in-phase contribution. Now, we assume those terms with a $\gamma$ factor are larger than the others and conclude that the *m* peaks are overall anti-phase with the $-\,m$ peaks but with unequal amplitudes. A large $\gamma$ corresponds to the long-lived state that was experimentally demonstrated in [12], and surprisingly the above model, which considers only the $\text {DQ}^{E_{\pm }}$ and the $\text {ZQ}^{E_{\pm }}$ transitions of the DD coupling between the collective spin and an external spin, results in an analytic solution that predicts most features of the NMR peaks that have been experimentally observed. Here, we are not interested in the exact dynamics of the spins system and focus on the permutation symmetry properties of spin operators of the Hamiltonian to obtain insightful understanding of the symmetry breaking mechanism. This is complimentary to the previous study [17] where the authors calculate the correlation function of the spherical top molecules explicitly and solve the master equation with the zeroth-order approximation numerically. In their approach, the nonzero matrix elements of the relaxation operator in the fully symmetric subspace are computed where in the limit of very fast rotation, the symmetry breaking components vanish. Our analytic study avoids the complexity of computing the explicit form of the spectral density of noise and avoids calculating large relaxation matrices and instead finds certain symmetry breaking patterns that lead to converting LLS into an NMR observable state. In both approaches, in a limit where the spectral density of noise is fully symmetric, LLS exhibits extremely long relaxation time.

### Relaxation induced by homonuclear coupling

In case of internal dipolar coupling, the analogy to Eq. 24 is

36 $$\begin{aligned} \frac{\partial \tilde{\rho }}{\partial t}= & {} \sum \limits _{\lambda } \ \mathbb {ZQ}^{\lambda }[\tilde{\rho }] + \mathbb {DQ}^{\lambda }[\tilde{\rho }] + \mathbb {SQ}^{\lambda }[\tilde{\rho }] \nonumber \\ \mathbb {ZQ}^{\lambda }[.]:= & {} \frac{1}{6}\ g^{\lambda }_{0}(0)\ \left( \hat{D}[ \mathbb {T}_{0}^{\lambda }][.] + \hat{D}[ \mathbb {T}_{0}^{-\lambda }][.] \right) \nonumber \\ \mathbb {DQ}^{\lambda }[.]:= & {} g^{\lambda }_{2}(2\ \omega _{s})\ \left( \hat{D}[\mathbb {T}_{2}^{\lambda }][.] + \hat{D}[\mathbb {T}_{-2}^{-\lambda }][.] \right) \nonumber \\ \mathbb {SQ}^{\lambda }[.]:= & {} g^{\lambda }_{1}( \omega _{s}) \ \left( \hat{D}[ \mathbb {T}_{1}^{\lambda }][.] + \hat{D}[ \mathbb {T}_{-1}^{-\lambda }][.] \right) . \end{aligned}$$

where

37 $$\begin{aligned} g^{\lambda }_{q}( \omega ):= \int \limits _{-\infty }^{\infty } \overline{G_{q}^{\lambda }(t) \ G_{q}^{\lambda *}(t+ \tau )} \hbox {e}^{-i \omega \tau } \hbox {d}\tau \end{aligned}$$

is the spectral density of noise due to the homonuclear dipolar Hamiltonian. We simply denote $g^{\lambda }_{q}( q\ \omega _{s})$ by $g^{\lambda }_{q}$.

We solve the master equation using the same approach we used in the previous section for heteronuclear coupling. In order to calculate the change of population of each energy level during the short evolution time $$\delta t$$, we need to know which transitions are allowed (the nonzero matrix elements of $$\mathbb {T}^{\lambda }_{q}$$ as shown in Fig. 2) and the initial imbalance of population between the allowed transitions. Thus, for each energy level $$ x \in \{|s, m\rangle \}$$, we should calculate $$\varDelta [x]_{ 1} = \sum \nolimits _{y} \ ([y]_{0} - [x]_{0}) \times W_{xy}$$ in which $$W_{xy}$$ are the transition rates and $$ [x]_{0}$$ is the population of the *x*th level at $$t=0$$.

For the initial state of interest (Eq. 27), at $t=0$, all the energy levels $| s, \frac{1}{2}\rangle$ have the same population as the $| s,- \frac{1}{2}\rangle$ energy levels. On the other hand, the transition rates induced by $\mathbb {ZQ}^{\lambda }$ terms of the internal dipolar coupling are identical for both $| s, \frac{1}{2}\rangle$ states and $| s, -\frac{1}{2}\rangle$. As a result, the change of population of $|s, m\rangle$ is equal to that of f $|s, -m\rangle$, leading to a zero polarization in the Zeeman basis. Therefore, even though there is an exchange of population between different symmetry subspaces, as shown in the middle section of Fig. 2, the net contribution of flip–flop interaction among protons to the NMR spectra of a protected state is zero.

Considering the above point, the solution of the master equation in Eq. 36 for a short evolution time, $\delta t$, is

38 $$\begin{aligned} \varDelta \left[ A, \pm \frac{3}{2}\right] _{1}= & {} \,- \frac{\delta t}{4} \ \left( C_{+}\ g_{1}^{E_{\mp }} + C_{-} \ g_{1}^{E_{\pm }}\right) \nonumber \\&-\, \delta \ t \left( C_{+}\ g_{2}^{E_{\mp }} + C_{-} \ g_{2}^{E_{\pm }}\right) \nonumber \\ \varDelta \left[ A, \pm \frac{1}{2}\right] _{1}= & {} \,- \frac{3\ \delta t}{4} \ \left( C_{+}\ g_{1}^{E_{\mp }} + C_{-} \ g_{1}^{E_{\pm }}\right) \nonumber \\ \varDelta \left[ E_{+}, \pm \frac{1}{2}\right] _{1}= & {} \,+ \ \frac{\delta t}{4} \ C_{+} \left( g_{1}^{E_{\mp }} + 3\ g_{1}^{E_{\pm }} \right) \nonumber \\&+\, \delta \ t\ C_{+} \ g_{2}^{E_{\pm }} \nonumber \\ \varDelta \left[ E_{-}, \pm \frac{1}{2}\right] _{1}= & {} \,+ \ \frac{\delta t}{4} \ C_{-} \left( g_{1}^{E_{\pm }} + 3\ g_{1}^{E_{\mp }}\right) \nonumber \\&+\, \delta \ t\ C_{-} \ g_{2}^{E_{\mp }} \end{aligned}$$

Given the above relations and $[x]_{\delta t}= [x]_{0} + \varDelta [x]_{1}$ for all $x \in \{ |s, m\rangle \}$, the expected NMR spectrum of protons for short evolution time is

$$\begin{aligned} \langle \mathbf {S}_{z} \rangle _{\delta t}= & {} \frac{3}{2}\ \left( \left[ A, \frac{3}{2}\right] _{\delta t} - \left[ A, -\frac{3}{2} \right] _{\delta t} \right) \\&+\, \frac{1}{2} \sum \limits _{s} \left( \left[ s, \frac{1}{2}\right] _{\delta t} - \left[ s, -\frac{1}{2} \right] _{\delta t} \right) \\= & {} \delta t \ (C_{+} - C_{-}) \ \left( \left( g_{1}^{E_{+}}- g_{1}^{E_{-}}\right) + 2\left( g_{2}^{E_{+}}- g_{2}^{E_{-}}\right) \right) \end{aligned}$$

We repeat the above calculation for the second time step, $t= 2\ \delta t$, using the method explained in “Appendix” and concluded that the signal is proportional to $(C_{+} - C_{-}) \ ( g_{q} ^{E_{+}} + g_{q}^{E_{-}})$. *This means that if the* $E_{+}$ and the $E_{-}$ *subspaces are equally populated at* $t=0$, *the higher-order contribution of the homonuclear dipolar relaxation to the NMR spectra is also zero*. We justify this counterintuitive result by comparing the spin operators of the heteronculear coupling to that of the homonuclear couplings. The $\text {ZQ}^{\lambda }$ and the $\text {DQ}^{\lambda }$ transitions of the external dipolar interaction occur when the protons of methyl group exchange energy with an external spin. This *effectively* appears as a single spin flip (or a $\mathbf {S}_{\pm }^{\lambda }$ operator) in the space of protons. In that sense the $T_{+}^{\lambda }$ of the homonuclear coupling is similar to the $\mathbf {S}_{\pm }^{\lambda }$ of the heteronuclear coupling. There are two important points that differentiate the two relaxation processes. The first point is that the $T_{+}^{\lambda }$ is block diagonal in the *E* subspace, meaning that there is no exchange of population between the $E_{+}$ and the $E_{-}$ subspaces as opposed to the $\mathbf {S}_{+}^{\lambda }$ operator. As a result, when these two spaces are equally populated at $t=0$, after an evolution time $\delta t$, the net polarization in the Zeeman basis remains zero, because both of these subspaces exchange population with the *A* subspace with the same rate but in opposite direction. Thus, the homonuclear coupling *passively* breaks the symmetry of an initially prepared protected state, meaning that even though it induces nonzero transition between different symmetry subspaces, its effective contribution to the NMR observation of LLS is zero. The second point is that, in case of heteronuclear interaction, when a $|s, m, \uparrow \rangle$ level experiences a $\text {DQ}$ transition with the rate $J_{2}^{\lambda }$ for instance, its counterpart $|s, -m, \uparrow \rangle$ experiences a $\text {ZQ}$ transition with the rate $\frac{1}{6}J_{0}^{\lambda }$. The differences between the two rates, $(J^{\lambda }_{2} - \frac{1}{6} \ J^{\lambda }_{0})$, appear as a nonzero factor in the NMR signal (Eq. ). This holds true even if the $E_{+}$ and the $E_{-}$ subspaces have identical initial population. Thus, the heterouclear coupling *actively* breaks the symmetry of an initially prepared protected state because it leads to a nonzero signal.

## Conclusion

We analyzed the contribution of both the internal and the external dipolar interactions of methyl groups in NMR observation of long- lived states that are initially prepared as symmetry-polarized states. The symmetry properties of spin operators and the density matrix allowed us to first show why these states exhibit long relaxation times and second to solve the master equation analytically and obtain the NMR spectrum. Our study provides insight into the nature of symmetry breaking interactions that lead to converting LLS into an NMR observable state and is in agreement with the reported experimental observations [12]. As complimentary to the previous study [17] where the authors solved the master equation by considering a particular correlation function, one can use the symmetry breaking patterns that we derived here to simplify the calculation of the master equation for any arbitrary spectral density of noise. We find that even though both the heteronuclear and homonuclear dipolar coupling break the symmetry of LLS, only the former leads to a relaxation pathway with nonzero polarization transfer from symmetry order to Zeeman order. This approach might find application in other areas in NMR where the underlying physics of the relaxation mechanisms is of interest.

## Appendix

In the main text, we solved the rate equations for a short evolution time, $t=\delta t$. To compute the change of population of the *x*th energy level during $t_{0}$ and $t_{0}+ \delta t$, it is required to know the population difference between the *x*th level and all other energy levels *y* at time $t_{0}$ which are allowed due to the $\text {DQ}^{\lambda }$ and $\text {ZQ}^{\lambda }$ terms of DD coupling. For the first step, when $t_{0}=0$, the calculation of population difference between allowed transitions yields Eq. 30 when the test spin is unpolarized and results in Eq. 34 when the test spin has a polarization $\alpha \ne 0$. In this appendix, we move one step forward and compute the change of population of the *x*th level during $t=\delta t$ and $t=2\delta t$. This requires the calculation of population difference between the allowed transitions, *x* and *y*, at an earlier time, $t_{0}=\delta t$. This is computed by

39 $$\begin{aligned}{}[x]_{\delta t}-[y]_{\delta t} = [x]_{0}-[y]_{0} + \varDelta [x]_{1}- \varDelta [y]_{1}. \end{aligned}$$

The first two terms, $$[x]_{0}-[y]_{0} $$, are already calculated in Eq. 34. To compute the second two terms, we assume $$\alpha =0$$, for simplicity, and use the result in Eq. 38. We start from $$m= \pm \frac{3}{2}$$ and obtain

40 $$\begin{aligned} \varDelta \left[ A, \frac{3}{2}, \uparrow \right] _{1}-\varDelta \left[ E_{\pm }, \frac{1}{2}, \downarrow \right] _{1}= & {} - \delta t\ \left( a_{0} + a_{\pm }\right) \nonumber \\ \varDelta \left[ A, \frac{3}{2}, \downarrow \right] _{1}-\varDelta \left[ E_{\pm }, \frac{1}{2}, \uparrow \right] _{1}= & {} - \delta t\ \left( b_{0} + b_{\mp }\right) \nonumber \\ \varDelta \left[ A,- \frac{3}{2}, \downarrow \right] _{1}-\varDelta \left[ E_{\pm }, -\frac{1}{2}, \uparrow \right] _{1}= & {} - \delta t\ \left( a_{0} + a_{\mp }\right) ^{\beta \rightarrow -\beta } \nonumber \\ \varDelta \left[ A,- \frac{3}{2}, \uparrow \right] _{1}-\varDelta \left[ E_{\pm }, -\frac{1}{2}, \downarrow \right] _{1}= & {} - \delta t\ \left( b_{0} + b_{\mp }\right) ^{\beta \rightarrow -\beta } \end{aligned}$$

in which $a_{0}$, $b_{0}$, $a_{\pm }$ $b_{\pm }$ are constants and the subscript $\beta \rightarrow -\beta$ means that in the definition of these constants, $\beta$ is replaced with $-\beta$. Those constants are explicitly

$$\begin{aligned} a_{0}= & {} \frac{\gamma }{4}\ \left( J^{E_{+}}_{2}+J^{E_{-}}_{2} + \frac{J^{E_{+}}_{0}+J^{E_{-}}_{0}}{18}\right) + \beta \frac{(1-\gamma )}{8} \ \left( J^{E_{+}}_{2}-J^{E_{-}}_{2}\right) \\ b_{0}= & {} \frac{\gamma }{4}\ \left( \frac{J^{E_{+}}_{0}+J^{E_{-}}_{0}}{6} + \frac{J^{E_{+}}_{2}+J^{E_{-}}_{2}}{3}\right) + \beta \frac{(1-\gamma )}{8}\ \left( J^{E_{+}}_{2}-J^{E_{-}}_{2}\right) \\ a_{\pm }= & {} \frac{\gamma }{4}\ \left( J^{E_{\pm }}_{2}- \frac{J^{E_{\pm }}_{0}}{18}\right) \pm \beta \frac{(1-\gamma )}{8}\ \left( \frac{J^{E_{\pm }}_{0}}{9}+ \frac{J^{E_{\mp }}_{0}}{18}\right) \\ b_{\pm }= & {} \frac{\gamma }{4}\ \left( \frac{J^{E_{\pm }}_{0}}{6}- \frac{J^{E_{\pm }}_{2}}{3}\right) \pm \beta \frac{(1-\gamma )}{8}\ \left( \frac{2J^{E_{\pm }}_{2}}{3}+ \frac{J^{E_{\mp }}_{2}}{3}\right) \end{aligned}$$

The expressions in Eq. 40 give the population difference between allowed transitions at time $t_{0}= \delta t$. Thus, we substitute Eq. 40 into the rate equation, Eq. 26, in order to calculate $\varDelta [x]_{2}$ and the result is

41 $$\begin{aligned} \varDelta \left[ A, \frac{3}{2}, \uparrow \right] _{2}= & {} \varDelta \left[ A, \frac{3}{2}, \uparrow \right] _{1} + \delta t^{2} \ \left( \left( a_{0} + a_{+}\right) \ J^{E_{+}}_{2} +\left( a_{0} + a_{-}\right) \ J^{E_{-}}_{2} \right) \nonumber \\ \varDelta \left[ A, \frac{3}{2}, \downarrow \right] _{2}= & {} \varDelta \left[ A, \frac{3}{2}, \downarrow \right] _{1} + \frac{\delta t^{2}}{6} \ \left( \left( b_{0} + b_{+}\right) \ J^{E_{+}}_{0} +\left( b_{0} + b_{-}\right) \ J^{E_{-}}_{0} \right) \nonumber \\ \varDelta \left[ A, -\frac{3}{2}, \uparrow \right] _{2}= & {} \varDelta \left[ A, -\frac{3}{2}, \uparrow \right] _{1} + \frac{\delta t^{2}}{6} \ \left( \left( b_{0} + b_{-}\right) \ J^{E_{-}}_{0} +\left( b_{0} + b_{+}\right) \ J^{E_{+}}_{0} \right) ^{\beta \rightarrow -\beta } \nonumber \\ \varDelta \left[ A, -\frac{3}{2}, \downarrow \right] _{2}= & {} \varDelta \left[ A, -\frac{3}{2}, \downarrow \right] _{1} + \delta t^{2} \ \left( \left( a_{0} + a_{-}\right) \ J^{E_{-}}_{2} +\left( a_{0} + a_{+}\right) \ J^{E_{+}}_{2} \right) ^{\beta \rightarrow -\beta }\nonumber \\ \end{aligned}$$

Then, to compute the NMR signal on the test spin channel condition on the collective spin magnetization $m=\pm \frac{3}{2}$, we subtract the above expressions from each other, and in the calculation, we encounter terms like this

$$\begin{aligned} (a_{0}+ a_{+}) J^{E_{+}}_{2} - (b_{0} + b_{+}) \frac{1}{6}J_{0}^{E_{+}} = \frac{\gamma }{4}\ A + \beta \frac{1- \gamma }{8}\ B \end{aligned}$$

which is written in such a way that *A* contains all terms with $\frac{\gamma }{4}$ dependency and *B* contains all terms with $\beta \frac{1- \gamma }{8}$. Intuitively, if $\beta =0$, the NMR signal from $\pm m$ has the same amplitude but with opposite sign. This is because the counter part of $|A\ ,\ +\frac{3}{2}, \uparrow \rangle$ is $|A\ ,\ -\frac{3}{2}, \downarrow \rangle$ where both are involved in $\text {DQ}$ transition and the counterpart of $|A, +\frac{3}{2}, \downarrow \rangle$ is $|A, -\frac{3}{2}, \uparrow \rangle$ where both are involved in $\text {ZQ}$ transition. Thus, the contribution from the $\frac{\gamma }{4}$-dependent terms results in an anti-phase signal from $m=\pm \frac{3}{2}$ peaks. In contrast, the contribution from the $\beta$-dependent terms results in an in-phase signal from $m=\pm \frac{3}{2}$ peaks, because, $m=-\frac{3}{2}$ picks an additional minus when $\beta \rightarrow -\beta$. Therefore, given the expressions in Eq. 41 and the first-order calculation that was given in the main text in Eq. , the second-order anticipated NMR signal for evolution time, $t=2\ \delta t$, is

$$\begin{aligned} \langle \varPi ^{\pm \frac{3}{2}} \otimes I_{z} \rangle _{2\delta t}= & {} \langle \varPi ^{\pm \frac{3}{2}} \otimes I_{z} \rangle _{\delta t} + \frac{\delta t^2}{8}\ \left\{ \pm \gamma \ A + \beta \frac{(1-\gamma )}{2}\ B \right\} \end{aligned}$$

The same logic holds for $m=\pm \frac{1}{2}$ NMR peaks, and similar feature is obtained,

$$\begin{aligned} \langle \varPi ^{\pm \frac{1}{2}} \otimes I_{z} \rangle _{2\delta t}= & {} \langle \varPi ^{\pm \frac{1}{2}} \otimes I_{z} \rangle _{\delta t} + \frac{\delta t^2}{8}\ \left\{ \pm \gamma \ A' + \beta \frac{(1-\gamma )}{2}\ B' \right\} \end{aligned}$$

It is important to note that in contrast to the calculated NMR signals for $t=\delta t$, here at $t=2 \delta t$, the NMR peaks of $m=\pm \frac{1}{2}$ do not have the same amplitude as that of $m=\pm \frac{3}{2}$. This has roots in the fact that at $t=\delta t$, all energy levels within each symmetry subspace are not equally populated any more, leading to $A\ne A'$ and $B\ne B'$.

To conclude, the solution of the rate equations for the second step, $t= 2\delta t$, has similar features to that of Eq. 31, and therefore, all arguments in the main text regarding the phase and amplitude of the NMR peaks, which were concluded for short evolution $t= \delta t$, can be generalized to any time evolution by inductive reasoning.

## Footnotes

1 In case of two identical spins, one can find a relation between the symmetry order and the thermal magnetization order [15].
